# Small size does not confer male agility advantages in a sexually-size dimorphic spider

**DOI:** 10.1371/journal.pone.0216036

**Published:** 2019-05-15

**Authors:** Shakira G. Quiñones-Lebrón, Matjaž Gregorič, Matjaž Kuntner, Simona Kralj-Fišer

**Affiliations:** 1 Evolutionary Zoology Laboratory, Biological Institute ZRC SAZU, Ljubljana, Slovenia; 2 Evolutionary Zoology Laboratory, Department of Organisms and Ecosystems Research, National Institute of Biology, Ljubljana, Slovenia; University of Richmond, UNITED STATES

## Abstract

Selection pressures leading to extreme, female-biased sexual size dimorphism (SSD) in spiders continue to be debated. It has been proposed that males of sexually size dimorphic spiders could be small because gravity constrains adult agility (locomotor abilities). Accordingly, small males should achieve higher vertical climbing speeds and should be more prone to bridge. The curvilinear model of the gravity hypothesis predicts a negative relationship between vertical climbing speed and male body size only over a threshold of 7.6 mm, 42.5 mg. Because males of most species with extreme SSD fall well below this threshold, the relationship between male size and agility at this scale remains vague. Here, we tested three hypotheses on how male size, mass and age (after maturation) relate to vertical climbing and bridging ability in *Nephilingis cruentata*, a highly sexually dimorphic orb-weaver with males well below the size threshold. We placed males of different sizes and adult ages in a vertical platform and recorded their climbing speeds. Contrary to the original study testing male bridging ability as binary variable, we measured the duration of the crossing of the bridging thread, as well as its sagging distance. Male body size and mass positively related to the vertical climbing speed and to the distance of the sagging thread during bridging, but had no influence on the bridging duration. The detected positive correlation between male size/mass and vertical climbing speed goes against our first prediction, that small males would have vertical climbing advantage in *Nephilingis cruentata*, but agrees with the curvilinear model. Against our second prediction, small males were not faster during bridging. Finally, in agreement with our third prediction, threads sagged more in heavier males. These results suggest that small male size confers no agility advantages in *Nephilingis cruentata*.

## Introduction

Among terrestrial animals, spiders (Araneae) exhibit the most extreme cases of female-biased sexual size dimorphism, i.e. females are much larger than males [1–3], with cases of females being up to 500 times heavier than males [1]. Despite considerable research efforts, the explanations for the evolution of sexual size dimorphism (SSD) in spiders remain controversial [4,5].

The most generally accepted explanation for female-biased SSD is the fecundity selection hypothesis proposing that the evolution towards larger females is driven by fecundity advantages [5–11]. While fecundity selection generally explains larger females, it remains unclear why males of sexually dimorphic spiders have persisted at small, ancestral sizes [7,12–14]. Several hypotheses attempting to explain the maintenance of small male sizes are based on life history differences between the sexes because in spiders, roving males are exposed to different selection pressures than are sedentary females. Among possible explanations for small male size is the differential mortality model that predicts a higher male mortality during mate search [15], but see [12]), which could result in small male size; the rationale is that a lowered male-male competition relaxes the selection for larger male size [16]. Other hypotheses aim to explain the small male size in relation to advantages in the contexts of ability to find mates [17,18] and mating opportunities [17,19,20], as well as the locomotor abilities of males during mate search [15,21,22], the focus of our study.

The gravity hypothesis (GH) [21] explains a greater female-biased SSD in those species whose males climb to reach mates in higher-ground habitats compared to those species that live close to the ground. The rationale for the GH is based on a biomechanical model predicting a negative relationship between male body size/mass and vertical speed [21]. If males’ vertical climbing speed was constrained by gravity (mass/size), small males would be faster, and more competitive in scramble competition or predator avoidance. This hypothesis has subsequently been questioned [23] because the majority of empirical studies have not supported its predictions (*Latrodectus hasselti* [24]; *Argiope aurantia* [25]; *L*. *hesperus* [26]; *Nephila plumipes* [17]; *Leucauge venusta* [27], *A*. *keyserlingi*, *N*. *plumipes*, *Jacksonoides queenslandica* [28]), but see *Trichonephila clavipes* [29]).

A modification of GH [30] suggests that the relationship between body size and climbing speed is not purely negative but curvilinear. Thus, a spider’s climbing speed relates negatively with size only above a certain size threshold. Below this threshold, plotted at 7.6 mm (and 42.5 mg), the relationship between size and speed would in fact be positive [30]. This optimal climbing-speed model proposes that “extreme SSD has evolved only in those species in which: (a) males have to climb to find females and (b) females are larger than the optimal climbing mass” [30]. The empirical evidence currently does not support this model [28].

A further refined gravity hypothesis (rGH) [22] proposes that larger spiders are less prone to bridge, i.e. cross empty spaces by the use of silken threads. Therefore, small male size would facilitate climbing, as well as bridging. However, a single empirical study on the crab spider *Misumena vatia* [31] showed a higher bridging speed in larger individuals, the opposite of the rGH predictions. Furthermore, a biomechanical model suggests that in species that use bridging for mate-search, larger individuals are limited by the increasingly sagging bridge thread [32]. However, this hypothesis has not been empirically tested.

**Fig 1:**
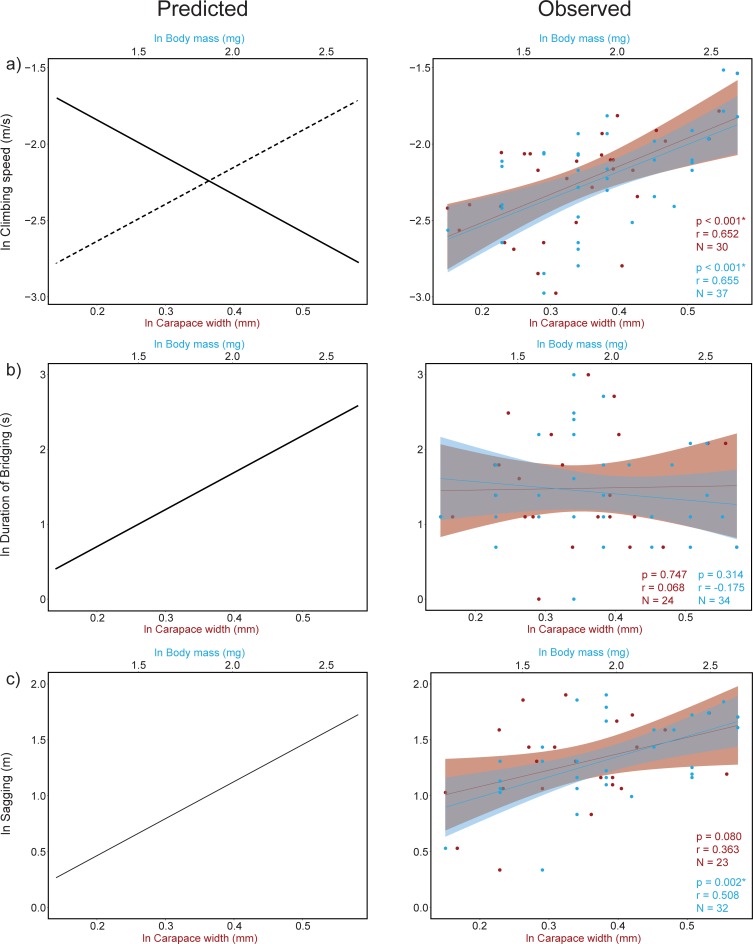
**Predicted (left) and observed (right) results.** (A) Both body size (in red) and mass (in blue) positively correlated to climbing speed. These results are against the prediction that small male size has a climbing advantage (black line), but fall within the predictions of the curvilinear model of the GH (dashed line). (B) Against our prediction, bridging duration did not correlate to either body size (in red) or mass (in blue). (C) Body mass (in blue), but not body size positively correlated to the sagging of the bridging thread.

Here, we investigate how vertical climbing and bridging speed vary with male body mass and size. We test these relationships in the African hermit spider (*Nephilingis cruentata*), an extremely sexually size dimorphic spider. With females 200 times heavier than males and a considerable male size (3–14 mg), this species is a suitable model to test for differential size advantages in locomotor abilities. Specifically, we explored the relationships among male physical traits (size, weight, condition, and senescence) and 1) climbing speed on vertical surfaces; 2) speed of bridge gaps in the air column, and 3) sagging bridge-threads. We predict a negative relationship between male body size/mass and climbing speed if smaller males have a climbing advantage, or alternatively, a positive relationship according to the curvilinear GH model (Fig 1A). We also predict a positive relationship between male size/mass and bridging duration (Fig 1B), as well as between male size/mass and the sagging of the bridging threads (Fig 1C).

## Materials and methods

### Species description and laboratory rearing

Hermit spiders, genus *Nephilingis*, are classified in the family Nephilidae that is known for the most extreme cases of SSD in spiders [33,34]. *N*. *cruentata* is a tropical African species with extreme SSD (female body size: 10–28 mm, male body size: 3.1–5.9 mm) [35]. Females generally construct aerial “semi-orb webs” such that there is a tubular retreat against a hard substrate (i.e. large trees and rocks) and an aerial orb [35]. *N*. *cruentata* females spend the daytime in their retreat and nights at the hub [35]. Males are observed cohabiting with females, generally mate inside their retreat, and break-off their palps during mating, thus becoming eunuchs [35]. Sexual cannibalism is common both before and during copulation.

Adult males of *N*. *cruentata* were obtained from the second generation of a laboratory population of the Evolutionary Zoology Lab ZRC SAZU in Ljubljana, Slovenia. The parental generation came from nine female lineages collected from iSimangaliso Wetland Park and Ndumo Game Reserve in South Africa (permit num. OP552/2015) between January and February 2015. The males were separated from the egg-sacs after their second moult and reared in an upside-down plastic cup (200mL) with a piece of cotton on the top to facilitate air and water exchange. They were sprayed with water daily, fed fruit flies (*Drosophila* sp.) ad libitum twice a week, and maintained in laboratory conditions where light/dark cycles and temperature were controlled (LD 12:12h, T = 25°C). Males moulted to maturation between 38 and 164 days, and were between one and 90 days old after maturation at the time of the experiment; the latter will be referred to as “senescence” for simplicity. Because our experimental males exhibited such a wide age range, we included senescence as a factor in our analyses. We selected senescence instead of total age as it is more biologically relevant because males from the same egg-sac differed greatly in life-histories as some spent much longer in the subadult stage. Carapace width was used as a linear measure of spider size since it remains constant once an individual has reached maturity [36]. For measuring carapace width, we photographed each spider using a Canon Eos 7D camera equipped with a Canon 50 mm macro lens. For all photography of spiders, we used a standardized fixed focal length and calculated carapace width by converting the pixel distance. Mass was measured using an electronic laboratory scale (KERN GI, 220-3NM; min = 0.02, d = 0.001g).

### The climbing experiment

To test maximum male climbing speed, we created a climbing platform consisting of a long piece of micropore tape (Micropore, Tosama) attached to a Perspex vertical surface (length = 60 cm). An adult male (N = 40) was placed at the bottom of the platform and encouraged to climb by gently touching his hind legs with a paintbrush (e.g. Brandt and Andrade). This elicits a response to predation, which motivates individuals to sprint [37]. The width of the micropore tape (25mm) allowed the males to climb up in a straight vertical direction while limiting walking in a horizontal direction. Each trial ended when the male reached the top of the platform. To precisely measure the climbing speed, the experiment was recorded with a stationary video camera at 60 frames per second. Each video was subsequently analysed with Tracker (https://physlets.org/tracker/) that automatically traced individual spiders and calculated their maximum speeds used as the measure of climbing speed to control for changes in individual motivation [28,38].

### The bridging experiment

**Fig 2:**
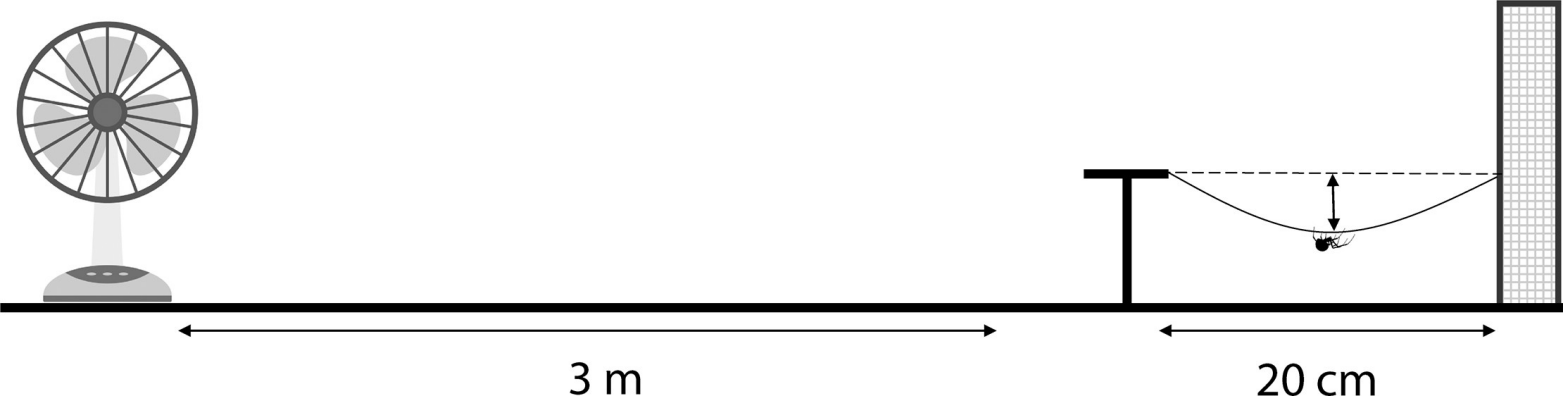
Scheme of the experimental set up for the bridging experiment. The male was placed over the T-shaped platform and allowed to bridge through a 20 cm gap to a landing platform made of a metal mesh that was held by a wooden frame. A low turbulence current was created using a desk fan at a 3m distance from the platform.

For the bridging experiment, adult males were placed on a t-shaped platform where they could hang from a safety thread and attempt to bridge to a landing platform. The landing platform was placed 20 cm away and consisted of a sheet of metal mesh attached to a wooden frame (Fig 2). A desk ventilator was placed 3 meters away and set to the lowest speed to create a low turbulent air flow. To observe bridging behaviour, we placed an adult male (N = 37; 31 males from the climbing experiment were reused) to the base of the T-shaped platform. Males climbed onto the edge of the platform and attempted to bridge to the mesh. As in the climbing experiments, males were recorded with a video camera at 60 frames per second, and the resulting videos were analysed using Tracker. We measured 1) the time it took for a spider to reach the platform once it started crossing the bridge and, 2) the difference in distance from an imaginary straight line between the silk thread’s anchor points and the spider as it sags while bridging.

### Statistical analyses

We checked data for normality using the Shapiro-Wilk test. Carapace width, body mass, senescence, and bridging duration were logarithmically transformed. Body condition was assessed as standardized residuals from regression with carapace width and body mass as independent and dependent variable, respectively. We used two approaches to test the relationships between males’ body measures and locomotor performance. In the first we correlated each body measure (carapace width, body mass, body condition) to each of the measured locomotion parameters (climbing speed, sagging distance, bridging duration) using Pearson correlations. The total number of samples in each correlation reflects the cases in which data were missing.

We then used linear regressions to model which of these body measures (predictors) best explain climbing speed, sagging distance, and bridging duration (outcome variables). Our explanatory variables showed multicollinearity due to high correlation between mass and carapace width (r = 0.828, N = 31, P > 0.001), therefore, we tested them separately against our response variables. For the first linear model we used carapace width and senescence as the predictor variables. The second model used mass, mass-independent structural size (i.e. residuals of size and mass regression), and senescence as the predictor variables.

For further testing, two values under the factor “sagging distance” were removed from the data after testing for outliers using the boxplot.stats function in R (R Developmental Core Team, 2014). The outlier values were a product of a breakage of the fibres in the silk thread, instead of natural variation.

## Results

We tested the climbing speed and the bridging ability on a total of 46 males with a high variability of body measures (Table 1). There was considerable variation in all the tested parameters of male ability in both climbing and bridging trials (Table 1).

**Table 1:** Summary of values of the independent and dependent variables.

	Median ± IQR	Min–max values
**Carapace width (mm)**	1.42 ± 0.20	1.16–1.78
**Body mass (mg)**	7.00 ± 5.00	3.00–14.00
**Senescence (days)**	65 ± 42	1–90
**Climbing speed (m/s)**	0.12 ± 0.04	0.05–0.22
**Bridging duration (s)**	4.00 ± 3.00	1.00–20.00
**Sagging distance (cm)**	4.40 ± 2.40	1.40–13.80

Bivariate correlations showed that climbing speed was positively correlated to carapace width and mass (carapace width, r = 0.652, N = 30, P < 0.001; mass, r = 0.655, N = 37, P < 0.001; Fig 1A), while negatively correlated with senescence (r = -3.67, N = 35, P = 0.001), but not significantly correlated to body condition (r = 0.231, N = 27, P = 0.238) (Table 2). The stepwise multiple regression of climbing speed against carapace width and senescence yielded a statistically significant model (adjusted R^2^ = 0.405, F_29,1_ = 21.42, P < 0.001) that included only carapace width as the significant predictor, accounting for 42% of the total variance. An additional stepwise multiple regression of mass against mass-independent size (i.e. residuals of regression between size and mass) and senescence yielded a statistically significant model (adjusted R^2^ = 0.4872, F_24,3_ = 9.55, P < 0.001) that included both mass and climbing senescence as the significant predictors, accounting for 54% of the total variance.

**Table 2:** Pearson correlation matrix of spider size and mass versus climbing and bridging variables. Significant results are bolded (P < 0.05).

	Body mass	Climbing speed	Bridging duration	Sagging distance
**Carapace width**	**r = 0.828**	**r = 0.652**	r = 0.068	*r = 0*.*365*
	**N = 31**	**N = 30**	N = 24	*N = 23*
	**P < 0.001**	**P < 0.001**	P = 0.747	*P = 0*.*080*
	**95% CI:** **(0.674, 0.913)**	**95% CI:** **(0.387, 0.817)**	95% CI: (-0.336, 0.451)	*95% CI*: *(-0*.*046*, *0*.*670)*
**Body mass**		**r = 0.655**	r = -0.175	**r = 0.508**
		**N = 37**	N = 34	**N = 32**
		**P < 0.001**	P = 0.314	**P = 0.002**
		**95% CI:** **(0.425, 0.806)**	95% CI: (-0.480, 0.168)	**95% CI:** **(0.120, 0.725)**
**Body condition**		r = 0.231	r = 0.171	r = -0.218
		N = 27	N = 22	N = 22
		P = 0.238	P = 0.433	P = 0.317
		95% CI: (-0.156, 0.556)	95% CI: (-0.259, 0.546)	95% CI: (-0.578, 0.213)
**Senescence**		**r = -3.67**	r = 0.273	r = -0.70
		**N = 35**	N = 31	N = 33
		**P = 0.001**	P = 0.118	P = 0.693
		**95% CI:** **(-0.790, -0.378)**	95% CI: (-0.072, 0.559)	95% CI: (-0.588, 0.054)

We found no relationships between bridging duration and any of the body measurements (body mass, r = -0.175, N = 34, P = 0.314; carapace width, r = 0.068, N = 24, P = 0.747; body condition, r = 171, N = 22, P = 0.433; Fig 1B) or senescence (r = 0.273, N = 31, P = 0.118).

The distance of the sagging bridging thread was significantly only affected by male mass (r = 0.508, N = 32, P = 0.002; Fig 1C) and accounted for 26% of the variance. No significant correlations were found between the sagging of the bridging thread and carapace width, body condition, or senescence (Table 2, Fig 1C).

## Discussion

Previous studies proposed that males of sexually size dimorphic spiders have persisted at small, ancestral sizes due to gravity that have limited adult agility over the size threshold of 7.6 mm and 42.5 mg. Namely, small male size should confer advantages in the contexts of vertical climbing and bridging. Yet, male size in the majority of those groups with extreme SSD lies below these thresholds and thus the relationship between male size and agility at this scale remains to be explored. We tested three hypotheses on whether and how male size, mass and senescence relate to vertical climbing and bridging ability in *Nephilingis cruentata*. We found that male body size and mass positively related to the vertical climbing speed and to the distance of the sagging thread during bridging, but had no influence on the bridging duration (Table 2, Fig 1). The detected positive correlation between male size/mass and vertical climbing speed goes against the prediction that small males would have vertical climbing advantage in *Nephilingis cruentata* but agrees with the prediction from the curvilinear GH [30] (Fig 1A). Against our prediction, small males were not faster during bridging (Fig 1B). Finally, in the line with our third prediction, threads sagged more in heavier males (Fig 1C). Our results thus imply that small male size confers no agility advantages in *Nephilingis cruentata*.

Our results on vertical climbing are consistent with the curvilinear GH, and the evidence found in the jumping spider *Jacksonoides queenslandica* [28,39], orb-weavers *Argiope keyserlingi* [39] and *A*. *aurantia* [40] where larger males, not smaller ones, were faster (see Fig 1A). Other studies, however, found no relationship at all between male size and climbing performance (*Latrodectus hesperus*, [26]; *Leucauge venusta*, [27]). Within the Nephilidae family, results are mixed; while there is some field evidence that *Trichonephila clavipes* males have a greater chance of arriving at female webs if they are smaller [29], the opposite is true for *T*. *plumipes* [17], and laboratory climbing tests in *T*. *plumipes* found that a male’s climbing speed positively correlated to body mass, but not with body size [28]. Currently, the evidence on relationship between male size and vertical climbing speed is inconclusive and fails to uniformly support the prediction that male sizes predict their vertical climbing abilities.

Vertical mobility of roving males can be affected by factors other than their mass or size, examples being substrate diameter (*A*. *keyserlingi*, *T*. *plumipes* [28]), body condition (*T*. *plumipes* [41]), and relative leg length (*J*. *queenslandica*, *T*. *plumipes* [41], *Argiope aurantia* [42]). Substrate diameter was controlled in our experiment; by using a 25 mm micropore tape, we created a wide substrate (i.e. slightly wider than leg span) under the assumption that males are more likely to climb wide surfaces (e.g. trees and human-made structures) in nature. Thus, our results should accurately reflect what would be the relative performance of males in nature. In *N*. *cruentata*, body condition had no effect on climbing speed, bridging duration, or sagging distance, which is in accordance with results found in *A*. *keyserlingi* and *J*. *queenslandica* [41]. However, we found a strong negative effect of senescence on climbing speed, which is expected, as physical ability normally declines with age [43]. We included senescence as a potential confounding factor, but the relationship between male size and climbing speed remained positive suggesting the robustness of size-vertical speed relationship in *N*. *cruentata* males. To our knowledge, no previous study had considered the effect of senescence in locomotor ability. This becomes particularly important when sampling randomly from natural populations, as in the case of the studies leading to the GH.

We further estimated the relationships between size/mass and locomotor abilities in the context of bridging. The rGH, proposing that larger spiders are less prone to bridge [22], is based on an experiment where bridging abilities were tested as binary variables (prone to bridge, not prone to bridge) in 13 species [22]. A binary response variable hinders the inference of relationship between size and bridging duration as a continuous variable, therefore we determined the effectiveness of bridging more precisely, as 1) the time it takes an individual to cross the gap after attaching the bridge thread, and 2) sagging distance of bridge thread. Against our second prediction however, male mass (gravity) did not affect bridging duration (Fig 1B). Namely, small males were not faster during bridging. Our result is inconsistent with the results from a study on *Misumena vatia*, where larger males were faster at bridging [31].

In agreement with our third prediction, we show that the sagging of the bridging thread is affected by the mass of the male (Fig 1C). Despite bridge threads of heavier males sagged more, they were not slower during bridging. Thus, it is possible that larger, heavier males compensate for bridge sagging through morphological adaptations [44,45], or by altering the use of silk tension and its properties [42,46–49]. The relationship between size and locomotor abilities in sexually size dimorphic spiders is likely more complex than suggested by the existing gravity hypotheses.

Overall, our results suggest that small male size confers no agility advantages in *N*. *cruentata*. According to the curvilinear model of GH and the rGH however, small size confers agility advantages only above the established size and mass thresholds. However, male sizes in the majority of extremely size dimorphic spider species (e.g. most Nephilidae, *Latrodectus*, *Argiope*, *Cyrtophora*, *Poltys*, *Arachnura*, *Caerostris*) are below these thresholds, and in some groups both sexes are below (e.g. *Acusilas*, *Deliochus*). Thus, we are left with no general explanation for why male size in those species is maintained over evolution. In other words, gravity might not be relevant for the evolution of extreme SSD in spiders.

## Supporting information

S1 File QuinonesetalDATA_2018. This is an Excel file with the data used for the analyses presented and discussed in this manuscript.
(XLSX)

S2 File RScript_Quinonesetal.R. This is an R script file with detailed data manipulation and analyses as presented in this manuscript. Note that it can only be opened in R.
(R) Click here for additional data file.
